# Insight into the structural stability of wild-type and histidine mutants in Pin1 by experimental and computational methods

**DOI:** 10.1038/s41598-019-44926-5

**Published:** 2019-06-10

**Authors:** Wang Wang, Lei Xi, Xiuhong Xiong, Xue Li, Qingyan Zhang, Wentao Yang, Linfang Du

**Affiliations:** Key Laboratory of Bio-resources and Eco-environment of the Ministry of Education, College of Life Sciences, Sichuan University, Chengdu, 610064 P.R. China

**Keywords:** Protein folding, Enzymes, Proteins

## Abstract

Pin1, a polypeptide proline isomerase parvulin, plays a key role in Alzheimer’s disease (AD), common tumors and cancers. Two conservative histidine residues, His59 and His157, are important for maintaining the stability of the PPIase domain. Hence multiple spectral and computational techniques were performed to investigate the potential mechanism of two histidine residues. Thermal denaturation indicated that both residues His59 and His157 are not sensitive to the lower temperatures, while residue His59 is more sensitive to the higher temperatures than residue His157. Acidic denaturation suggested that influences of both residues His59 and His157 to acidic stability were the difference from Pin1-WT. ANS and RLS spectra hinted that there was no significant effect on hydrophobic change and aggregation by histidine mutations. The GndHCl-induced denaturation implied that residues His59 and His157 contributed the most to the chemical stability. MD simulations revealed that residues His59 and His157 mutations resulted in that the hydrogen bond network of the dual histidine motif was destroyed wholly. In summary, these histidine residues play an important role in maintaining the structural stability of the PPIase domain.

## Introduction

Pin1 (Protein interaction with NIMA1), which was discovered in 1996, is a peptidyl-prolyl *cis-trans* isomerase (PPIase)^1^. Indeed, Pin1 is associated with many biological processes including transcription regulation, cell growth, cell division, cell cycle, apoptosis and DNA damage repair^2–4^. Moreover, Pin1 interacts with a number of phosphoproteins, such as c-Jun, cyclin D1, p53, tau protein and β-catenin, to modulate their structures and functions^3,5–8^. Interestingly, the changes of Pin1 activity are closely related to the types of disease. For example, the up-regulation of Pin1 activity leads to various cancers, on the contrary, it causes Alzheimer’s disease^2,3,5,9,10^. The stability of Pin1 plays a key role in performing its normal physiological activities and functions, hence researching the factors that affect the stability of Pin1 will be conducive to the diagnosis and treatment of Pin1-related diseases^10,11^.

**Figure 1:**
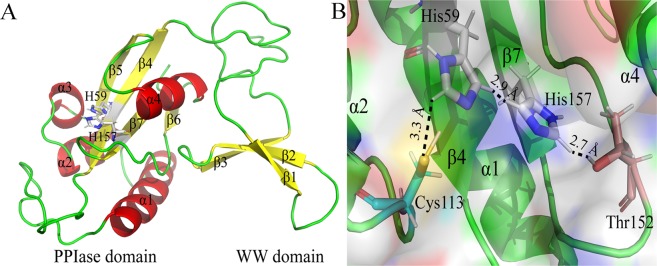
The three-dimensional structure of Pin1. (**A**) The Overall crystal structure of Pin1. Yellow arrows, green lines and red helixes represent β-sheet, random coil and α-helix, respectively. The model of white sticks represents residues His59 and His157, respectively. (**B**) The dual-histidine motif of Pin1. The model of white, blue-green and pink sticks represents histidine, cysteine and threonine residues, respectively. The black line represents hydrogen bonds.

Pin1 contains 163 amino acids and consists of two domains, the WW and PPIase domain, connected by a long flexible loop^12,13^ (Fig. 1A). The WW domain, which contains two highly conserved tryptophan residues, possesses a substrate identification pocket surrounded by the residues Ser16, Arg17, Tyr23 and Trp34^11,14^. The PPIase domain is a catalytic domain to with the function isomerize the peptides interacting with the residues Lys63, Arg68, Arg69, Cys113 and Ser154^7,14^. Moreover, two highly conserved histidine residues His59 and His157, within the active site, form a catalytic tetrads with the residues Cys113 and Thr152^15,16^ (Fig. 1B). Although their sequences are conservative in the PPIase domain, the role of these histidine residues remains unclear in Pin1.

Moreover, mutational analyses have already reported that several key amino acid residues are of great significance in Pin1, such as the residues Lys13, Trp11 and Ser32, they have played an important role in maintaining the structure and function of WW domain^11,17^. Similarly, the residues Val55, Cys57, Trp73, Leu61, Gly120, Ala137 and Gly155 have illustrated that they are essential to the structural stability of the PPIase domain^6,11,17^. Interestingly, the residues Leu61, Lys63, Ser67, Arg74 and Ala85 have possessed the function of phosphate binding^6,17^, and the residues Ser111 and Cys113 have owned the role of catalysis^6,17^. In addition, previous research has indicated that residues His59 and His157 are very vital in sustaining the domain structure or stability of Pin1^15,16^. In the present research, the site-directed mutation of histidine to arginine was constructed, and the spectral methodologies and molecular dynamics (MD) simulations were performed to investigate the structural stability of the PPIase domain. In conclusion, the present work will contribute to further understand the mechanism by which dual histidine motif maintain the stability of the PPIase domain.

## Results

### Thermal stability of Pin1-WT, H59R and H157R

**Figure 2:**
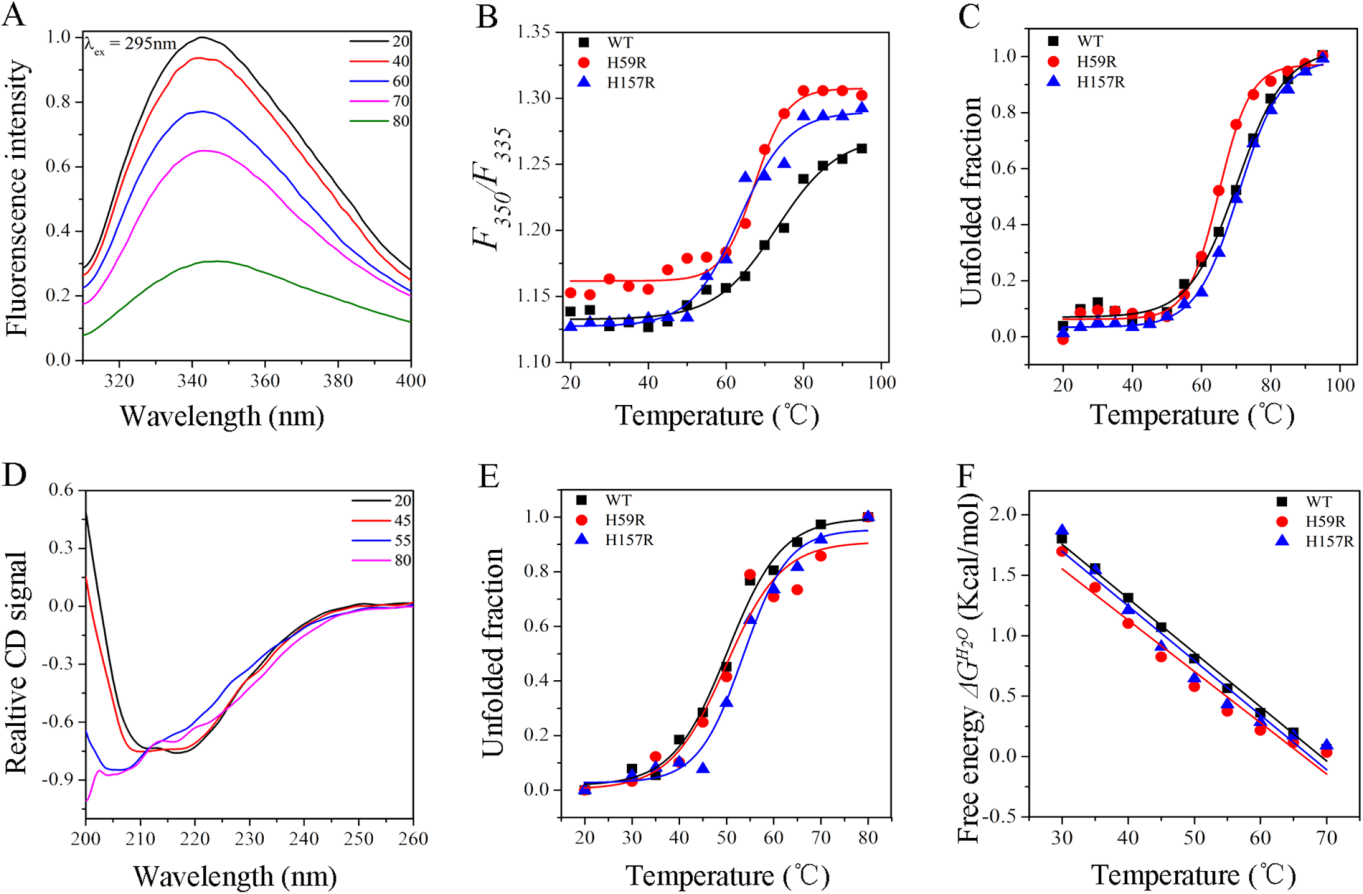
The thermal unfolding curves of Pin1-WT, H59R and H157R. (**A**) The representative fluorescence spectra of Pin1-WT. (**B**) The structural changes in the *F*_350_*/F*_335_ ratio for the spectra of Pin1-WT, H59R and H157R. (**C**) The thermal unfolding curves of Pin1-WT, H59R and H157R on the basis of fluorescence spectra with λ_ex_ = 295 nm. (**D**) The representative far-UV CD spectra of Pin1-WT. (**E**) The relative intensity of far-UV CD spectra of Pin1-WT, H59R and H157R at 208 nm. (**F**) The thermal unfolding curves of Pin1-WT, H59R and H157R on the basis of far-UV CD spectra.

The fluorescence spectra can reflect accurately the information of tertiary structure and hydrophobic change concerning the microenvironments around the chromophore^18,19^. Generally, the spectra with λ_ex_ = 295 nm (λ_ex_ = excitation wavelength) reveal the microenvironments around the tryptophan residues^20,21^. As shown in Fig. 2A, the fluorescence intensity of Pin1-WT decreased gradually with the increase of temperature, data of mutants not shown, which indicated that the polarity around the tryptophan residues gradually increased in the unfolded process. Ordinarily, the *F*_350_*/F*_335_ ratio was used to reflect the changes in the λ_max_ of the fluorescence spectra (λ_max_ = max emission wavelength)^11,22^. The increase of *F*_350_*/F*_335_ ratio meant red-shift of λ_max_, conversely, the decrease of the ratio indicated blue-shift of λ_max_. The shifts of *F*_350_*/F*_335_ ratio (Fig. 2B) had significant differences between Pin1-WT and mutants, suggesting that histidine mutations have an impact on the thermal stability of the protein structure. In addition, the intermediate states were obviously observed at about 60 °C by normalized data of Pin1-WT, H59R and H157R, which suggested that they involve a three-state transition during thermal denaturation process^20,21^. Therefore, the thermal unfolding of Pin1-WT and mutants was fitted to two-step denaturation, from 20 to 60 °C and 60 to 95 °C, in order to calculate the unfolded fraction of each protein on the basis of the fluorescence intensity at 340 nm using Eq. (2) (Fig. 2C).

*T*_*m*_, a criterion for the thermostability, defined as the midpoint of the denaturation process^11,23^, was summarized in Table 1. On the one hand, the *T*_*m*_ values of Pin1-WT, H59R and H157R were similar when the temperature was between 20 and 60 °C, indicating that the influence of histidine mutations to structural stability was not sensitive below 60 °C. On the other hand, the *T*_*m*_ value of H59R was the lowest and the others were similar, when the temperature was between 60 and 95 °C, suggesting that residues His59 was more sensitive to high temperature. Previous research has illustrated that histidine residues His59 and His157 play a significant role in structural stability, and His157 is not key in Pin1 function as His59^15^. Therefore, we deduced that the influences of residues His59 and His157 to the thermostability were similar Pin1-WT when the temperature was below 60 °C, while the influence of residue His59 to the thermostability was more sensitive when the temperature is above 60 °C.

**Table 1:** Fitted parameters for the thermal unfolding of Pin1-WT, H59R and H157R.

Thermal denaturation	*T*_*m*_ (^*o*^*C)*			$\Delta {{G}}^{{{H}}_{{2}}{O}}$ (Kcal mol^−1^)	*m* (Kcal mol^−1^ M^−1^)
	Fluorescence		Far-UV CD		
WT	54.57 ± 1.23	71.54 ± 0.46	53.74 ± 0.79	3.10 ± 0.07	0.044 ± 0.001
H59R	55.75 ± 4.81	60.59 ± 4.48	50.31 ± 0.87	2.83 ± 0.16	0.042 ± 0.003
H157R	54.50 ± 4.09	68.76 ± 1.26	50.02 ± 1.03	3.05 ± 0.18	0.045 ± 0.004

*T*_*m*_, the temperature of the denaturation process at the midpoint.

$\Delta {{G}}^{{{H}}_{{2}}{O}}$, the free energy of unfolding.

*m*, the experimental measure of the dependence of *ΔG*_*u*_ on temperature, respectively.

^+^The two-step transitions were used to analyze the unfolding of Pin1-WT, H59R and H157R, from 20 to 60 °C and from 60 to 95 °C.

The far-UV CD spectra can provide accurately the information of secondary structure^11,19,23^. The representative far-UV CD signal of Pin1-WT showed in Fig. 2D. The CD signal revealed that heat treatment changed the secondary structure of Pin1, resulting in the decreases of α-helix structure and increases of β-sheet and turn structure, respectively (Fig. 2D and Table S1). The unfolded fractions of Pin1-WT and mutants were plotted in Fig. 2E on the basis of the signal intensity at 208 nm. The free energies of unfolding $(\Delta {{G}}^{{{H}}_{{2}}{O}})$ were obtained by using the Eqs (3) and (4) (Fig. 2F). The one-step denaturation was applied to the unfolding of Pin1-WT and mutants by far-UV CD spectra and summarized in Table 1. *T*_*m*_ value of Pin1-WT was about 54 °C, while the histidine mutations resulted in that *T*_*m*_ values decrease by 3 °C, suggesting that histidine residues play an important role in maintaining the secondary structural stability of Pin1. The free energies of unfolding $(\Delta {{G}}^{{{H}}_{{2}}{O}})$ for Pin1-WT, H59R and H157R were 3.10, 2.83, 3.05 kcal mol^−1^, respectively, suggesting that H59R was relatively sensitive to heat and less thermostability than the others.

### Acidic stability of Pin1-WT, H59R and H157R

**Figure 3:**
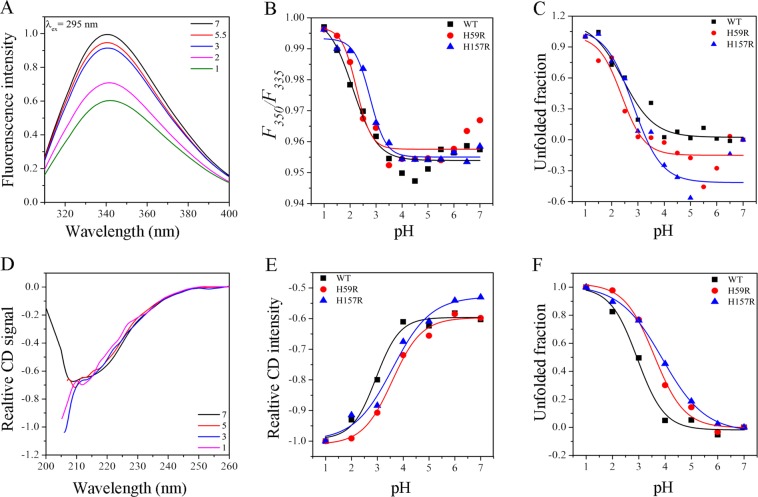
The acidic unfolding curves of Pin1-WT, H59R and H157R. (**A**) The representative fluorescence spectra of Pin1-WT. (**B**) The structural changes in the *F*_350_*/F*_335_ ratio for the spectra of Pin1-WT, H59R and H157R. (**C**) The acidic unfolding curves of Pin1-WT, H59R and H157R on the basis of fluorescence spectra with λ_ex_ = 295 nm. (**D**) The representative far-UV CD spectra of Pin1-WT. (**E**) The relative intensity of far-UV CD spectra of Pin1-WT, H59R and H157R at 208 nm. (**F**) The acidic unfolding curves of Pin1-WT, H59R and H157R on the basis of far-UV CD spectra.

The Fig. 3A showed the fluorescence spectra of Pin-WT by acidic treatment, data of mutants not shown. We observed that the fluorescence intensity decreased gradually in the acidic denaturation process with the decrease of acidic pH. The *F*_350_*/F*_335_ ratio was performed in the study of acid stability for Pin1-WT and mutants^22,24^. As shown in Fig. 3B, the *F*_350_*/F*_335_ ratio changed barely from pH 7.0 to pH 3.0, however, the ratio increased dramatically from pH 3.0 to pH 1.0. These phenomena elaborated that the acidic denaturation of Pin1-WT and mutants was a three-state transition and existed an intermediate, which are in accordance with the previous studies^24^. Therefore, the acidic denaturation of Pin1-WT and mutants was fitted to two-step denaturation, from pH 7.0 to pH 3.0 and pH 3.0 to pH 1.0. In order to calculate the unfolded fraction of protein on the basis of the fluorescence intensity at 340 nm, Eq. (2) was applied to fit data (Fig. 3C and Table 2).

**Table 2:** Fitted parameters for the acidic unfolding of Pin1-WT, H59R and H157R.

Acidic denaturation	p*K*_*a*_			p*K*_*b*_	p*K*_*c*_
	Fluorescence		Far-UV CD	ANS	RLS
WT	3.87 ± 0.27	2.43 ± 0.28	2.94 ± 0.14	4.50 ± 0.10	3.21 ± 0.04
H59R	4.22 ± 0.88	2.37 ± 0.27	3.48 ± 0.13	4.43 ± 0.07	3.27 ± 0.09
H157R	3.90 ± 0.59	2.82 ± 0.34	3.54 ± 0.08	4.42 ± 0.08	3.25 ± 0.05

p*K*_*a*_, the pH of the denaturation process at the midpoint.

p*K*_*b*_, the pH of one half of full exposure by ANS spectra.

p*K*_*c*_, the pH of the maximum intensity at 450 nm by RLS spectra.

^+^The two-step transitions were used to analyze the unfolding of Pin1-WT, H59R and H157R, from pH 7.0 to pH 3.0 and from pH 3.0 to pH 1.0.

p*K*_*a*_, the midpoint pH value in the unfolding process, is a criterion for the acidic stability of the protein structure^25,26^. For one thing, the p*K*_*a*_ value of H59R was the highest, and the others was similar, when pH was between 7.0 and 3.0, suggesting that residue His59 was relatively sensitive to acid. For another, the opposite results were observed that the p*K*_*a*_ value of H157R was the highest when pH is between 3.0 and 1.0. These results indicated that influences of both residues His59 and His157 to acidic stability were difference from Pin1-WT.

The representative far-UV CD signal of Pin-WT, data of mutants not shown, was plotted in Fig. 3D. The CD signal showed two typical negative bands at 208 and 217 nm in a native state. However, the acidic treatment changed greatly the shape of CD spectra, resulting in that the negative band at 217 nm faded away with the decrease of acidic pH^11,24^. In addition, the signal changes at 208 nm of Pin-WT and mutants decreased gradually from pH 7.0 to pH 1.0, suggesting the decrease of α-helix structures (Fig. 3E and Table S2). The unfolded fractions were showed in Fig. 3F on the basis of the signal intensity at 208 nm. The one-step denaturation was performed to the unfolding of Pin1-WT and mutants by far-UV CD spectra^11^. As seen in Table 2, the p*K*_*a*_ value of Pin-WT was the lowest, suggesting mutants were more sensitive to acid. These results further illustrated that the histidine residues are very important to the secondary stability of Pin1.

**Figure 4:**
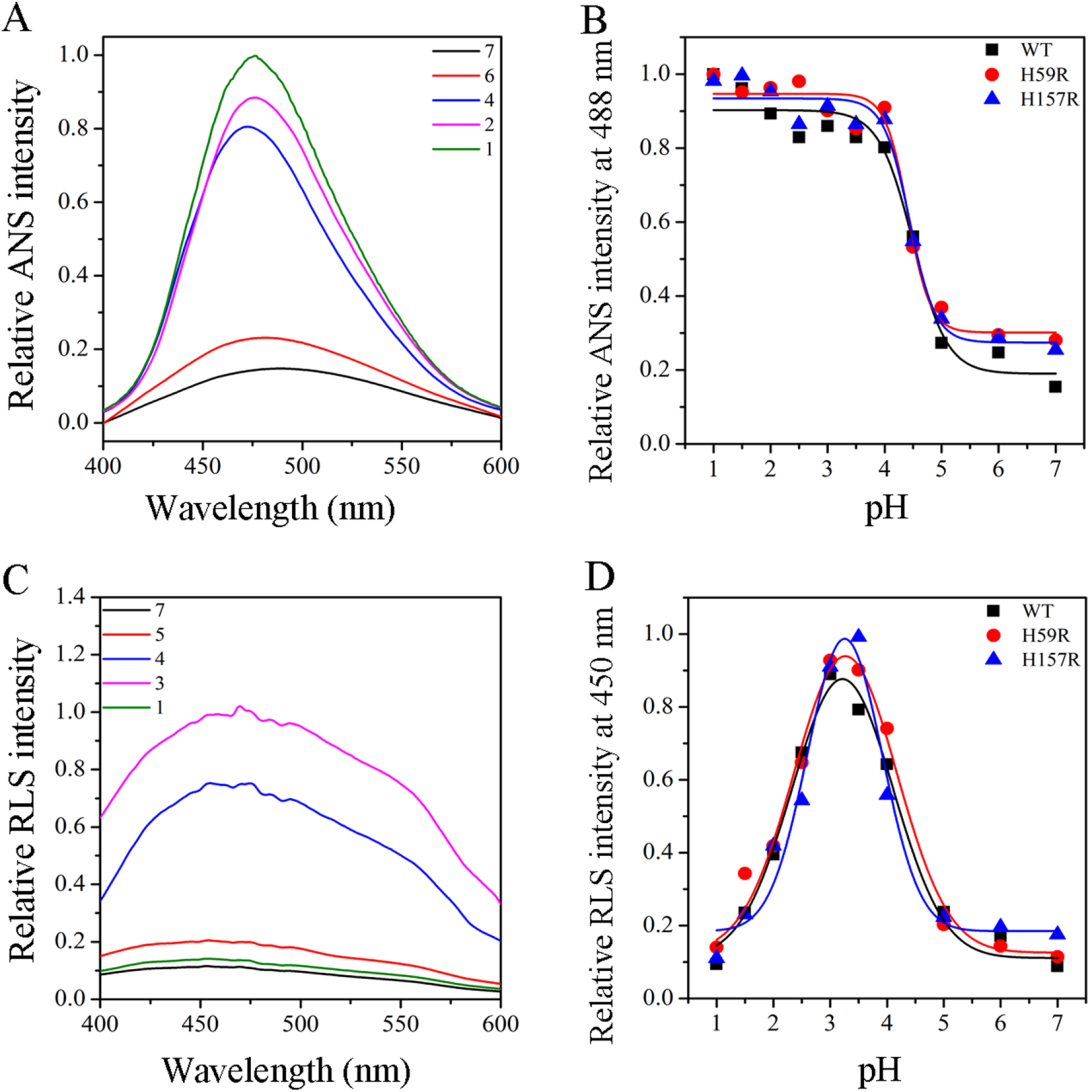
The ANS and RLS spectra of Pin1-WT, H59R and H157R by acidic pH treatments. (**A**) The representative ANS fluorescence spectra of Pin1-WT. (**B**) Relative ANS intensity at 488 nm of Pin1-WT, H59R and H157R. (**C**) The representative RLS fluorescence signal of Pin1-WT. (**D**) Relative RLS intensity at 440 nm of Pin1-WT, H59R and H157R.

The ANS fluorescence spectra were carried out to assess the exposure of the hydrophobic regions in Pin1-WT and mutants^22,24^. The representative ANS spectra of Pin-WT, data of mutants not shown, was plotted in Fig. 4A. Similarly, the fluorescence intensity at 488 nm was showed in Fig. 4B. These facts illustrated that ANS intensity was very weak from pH 7.0 to pH 5.0, while it increased dramatically from pH 5.0 to pH 3.0 and remained constant from pH 3.0 to pH 1.0. To calculate the hydrophobic parameters by fitting data, p*K*_*b*_ is the pH of one half of full exposure by ANS spectra (Table 2). The p*K*_*b*_ value of Pin1-WT was in accordance with these mutants at about pH 4.5, implying that the histidine residues had a little effect on the acidic pH-induced hydrophobic change.

The RLS fluorescence spectra are usually implemented to evaluate the aggregation and size of Pin1-WT and mutants in unfolding process^19,24^. The representative RLS spectra of Pin-WT and the fluorescence intensities were shown in Fig. 4C,D, respectively. p*K*_*c*_, which is defined as the pH of the maximum RLS fluorescence intensity at 450 nm, was summarized in Table 2. The p*K*_*c*_ value of Pin1-WT was in accordance with these mutants at about pH 3.3, indicating that the histidine residues to the acidic pH-induced aggregation were not a significant influence.

### Chemical stability of Pin1-WT, H59R and H157R

**Figure 5:**
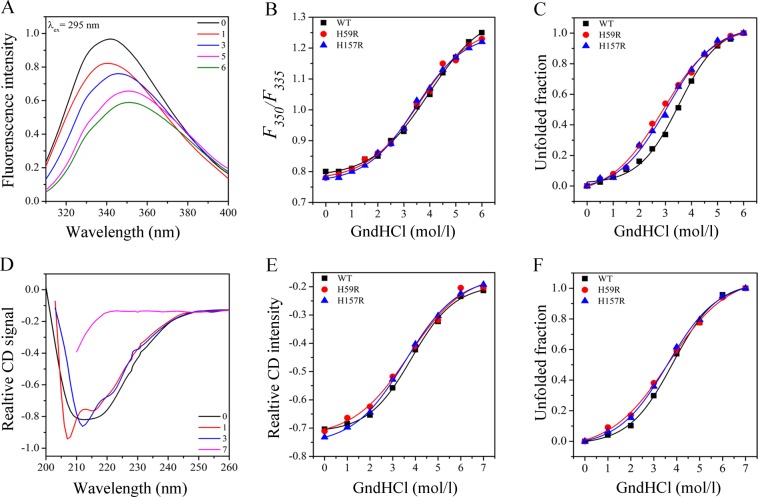
The chemical unfolding curves of Pin1-WT, H59R and H157R. (**A**) The representative fluorescence spectra of Pin1-WT. (**B**) The structural changes in the *F*_350_*/F*_335_ ratio for the spectra of Pin1-WT, H59R and H157R. (**C**) The chemical unfolding curves of Pin1-WT, H59R and H157R on the basis of fluorescence spectra with λ_ex_ = 295 nm. (**D**) The representative far-UV CD spectra of Pin1-WT. (**E**) The relative intensity of far-UV CD spectra of Pin1-WT, H59R and H157R at 222 nm. (**F**) The chemical unfolding curves of Pin1-WT, H59R and H157R on the basis of far-UV CD spectra.

The representative fluorescence spectra of Pin-WT, data of mutants not shown, was plotted in Fig. 5A by GndHCl treatment. The λ_max_ of native Pin1-WT and mutants were located at about 342 nm, while the GndHCl denaturation shifted λ_max_ to about 350 nm, indicating that the tryptophan residues in Pin1-WT and mutants were fully exposed after GndHCl denaturation. The *F*_350_*/F*_335_ ratio was used in GndHCl induced denaturation for Pin1-WT and mutants^11^. As shown in Fig. 5B, the *F*_350_*/F*_335_ ratio has hardly changed in the GndHCl of low concentration (<2.0 mol/l), however, the ratio increased significantly with the increase of GndHCl concentration. The unfolded fraction of each protein was showed in Fig. 5C. *C*_*m*_, the unfolding transition midpoint concentration, is a measure for the chemical stability of protein structure^11,19^. Obviously, the unfolding curves and *C*_*m*_ values of these histidine mutants were significantly different from Pin1-WT, suggesting that the residues His59 and His157 contribute to the chemical stability of Pin1 (Table 3).

**Table 3:** Fitted parameters for the chemical unfolding of Pin1-WT, H59R and H157R.

GndHCl denaturation	*G*_*m*_ (M)		*ΔG*_0_ (Kcal/mol)		*log K*_*obs*_	
	Fluorescence	Far-UV CD	Fluorescence	Far-UV CD	Fluorescence	Far-UV CD
WT	3.51 ± 0.06	3.84 ± 0.07	2.54 ± 0.09	2.70 ± 0.14	−1.86 ± 0.06	−1.97 ± 0.10
H59R	2.88 ± 0.07	3.66 ± 0.10	2.23 ± 0.10	2.30 ± 0.10	−1.63 ± 0.10	−1.68 ± 0.07
H157R	3.04 ± 0.06	3.61 ± 0.06	2.31 ± 0.08	2.05 ± 0.12	−1.69 ± 0.08	−1.50 ± 0.09

*C*_*m*_, the denaturant concentration of GdnHCl at the transition midpoint.

*ΔG*_0_, the free energy of unfolding.

*log K*_*obs*_, the unfolding kinetics rate constants.

The representative far-UV CD signal of Pin-WT, data of mutants not shown, was plotted in Fig. 5D by GndHCl treatment. With the increasing concentration of GdnHCl, the CD spectra were greatly changed and negative bands at 222 nm faded away, indicating the decreases of α-helix structure and increases of β-sheet and turn structure (Table S2). Moreover, the relative CD intensity at 222 nm decreased to the minimum at 7.0 M GdnHCl, implying complete denaturation of Pin1-WT and mutants (Fig. 5E). The unfolding curves of H59R and H157R were similar and their *C*_*m*_ values were less than Pin1-WT, meaning that histidine mutants were sensitive to the GdnHCl (Fig. 5F and Table 3). These phenomena were consistent with thermal and acidic denaturation by CD spectra, indicating that the histidine residues play a significant role in maintaining the secondary stability.

**Figure 6:**
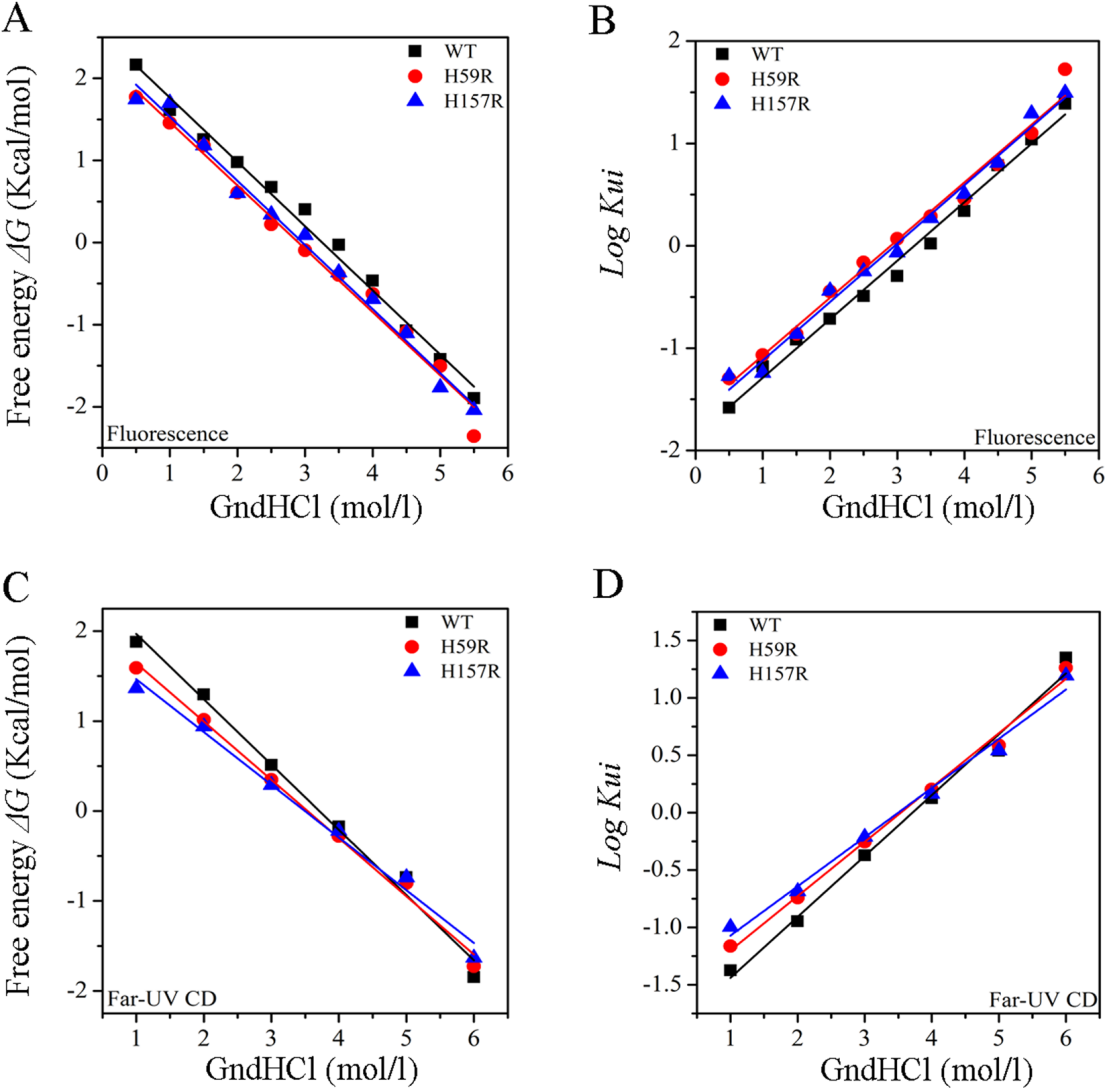
Chemical-induced conformational changes. (**A**,**B**) Represent free energy of unfolding (*ΔG*_0_) and unfolding kinetics rate constants (*log K*_*ui*_) against GdnHCl on the basis of fluorescence spectra, respectively. (**C**,**D**) Represent free energy of unfolding (*ΔG*_0_) and unfolding kinetics rate constants (*log K*_*ui*_) against GdnHCl on the basis of far-UV CD spectra, respectively. Intercepts at y axis give *ΔG*_0_ and *log K*_*obs*_, respectively.

The free energies of unfolding (*ΔG*_0_) and rate constants of unfolding (*K*_*obs*_) in Pin1-WT and mutants were calculated by Eqs (5) and (6), respectively^7,20^. The *ΔG*_0_ of H59R and H157R were similar and greater than Pin1-WT as revealed by fluorescence and far-UV CD spectra, suggesting that these histidine mutations is conducive to unfolding for Pin1 (Fig. 6A,C and Table 3). The *K*_*obs*_, which reflect the rate of unfolding, also illustrated that histidine mutations have an influence on the unfolding rate (Fig. 6B,D and Table 3).

### Overall dynamics behavior of the Pin1-WT, H59R and H157R

**Figure 7:**
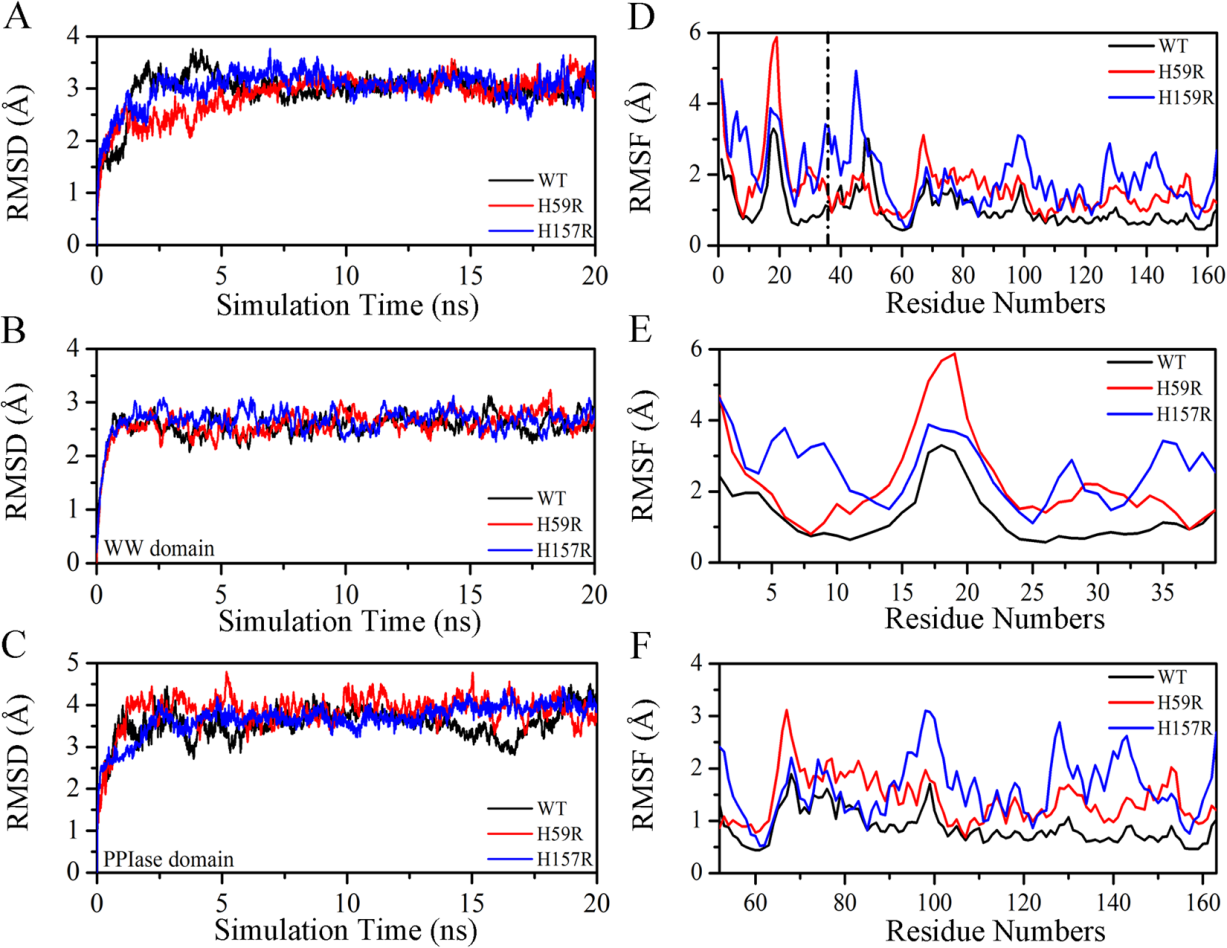
The RMSD and RMSF results in Pin1-WT, H59R and H157R as a function of 20 ns MD simulation. (**A**–**C**) Represent Cα**-**RMSD of the overall structure, PPIase and WW domain, respectively. (**D**,**E**) Represent Cα**-**RMSF of the overall structure, PPIase and WW domain, respectively.

It is well known that structural stability and flexibility can be altered by mutation methods^27,28^. Therefore, Cα-RMSD and Cα-RMSF were plotted in Fig. 7 to assess structural stability and flexibility, respectively. In Fig. 7A, the global structure Cα-RMSD values for Pin1-WT and mutants reached equilibrium after 5 ns with an average Cα-RMSD value of 3 Å. Previous researches have demonstrated that the movement of the WW and PPIase domain was relatively independent, so we calculated Cα-RMSD values of the WW and PPIase domain, respectively^28^. As shown in Fig. 7B, the WW domain of Pin1-WT and mutants shared similar RMSD values, indicating that the effect of histidine mutations was very weak for WW domain. However, there were obvious differences in the PPIase domain between Pin1-WT and mutants, with 3.54, 3.67, 3.85 Å of the average RMSD values (Table S4), respectively. The results further indicated that residues His59 and His157 have an effect on the stability of PPIase domain due to these histidine residues were located in this region (As seen in Fig. 1A). Bailey *et al*. works revealed that the dual histidine motif (His59 and His157) has contribution to structural stability rather than function in Pin1^15^. Combined with the results above, our work also implied the similar result about the influence of residues His59 and His157 to the PPIase domain of structural stability in the PPIase domain.

Usually, Cα-RMSF value provides the information for the structural flexibility and mobility of each protein^22,27^. Compared with Pin-WT, H59R and H157R may have an influence on the overall structural flexibility and mobility, with the average RMSF values increased by 0.59 and 0.92 Å, respectively, (Fig. 7D and Table S4). As seen in Fig. 7E, the significant difference of Cα-RMSF values occurred at residues 15–25 in the WW domain, which consist of a flexible loop between β1 and β2. In Fig. 7F, H59R had an impact on the RMSF values of the residues 67–72, which are located at a functional loop to bind substrates. However, H157R affected both the RMSF values of residues 95–103 and 125–145, which are located at α1/α2 and α4 structure (Fig. 1A). Generally, the active center of Pin1 in the PPIase domain is composed of residues Lys63, Arg68, Arg69, Cys113 and Ser154^12,13^. Therefore, we conjectured that the residue His59 might affect the structural flexibility and mobility of active center in Pin1 due to its spatial position is close to the key catalytic residues Cys113^15,29^. Moreover, the hydrophobic pocket are mainly composed of residues His59, Ser114, Ala116, Leu122, Gln129, Gln131, Phe134 and His157 in the PPIase domain^14,30^. We guessed that H157 affects possibly the structural flexibility and mobility of the hydrophobic pocket.

### Analysis of distance and Rg in the Pin1-WT, H59R and H157R

**Figure 8:**
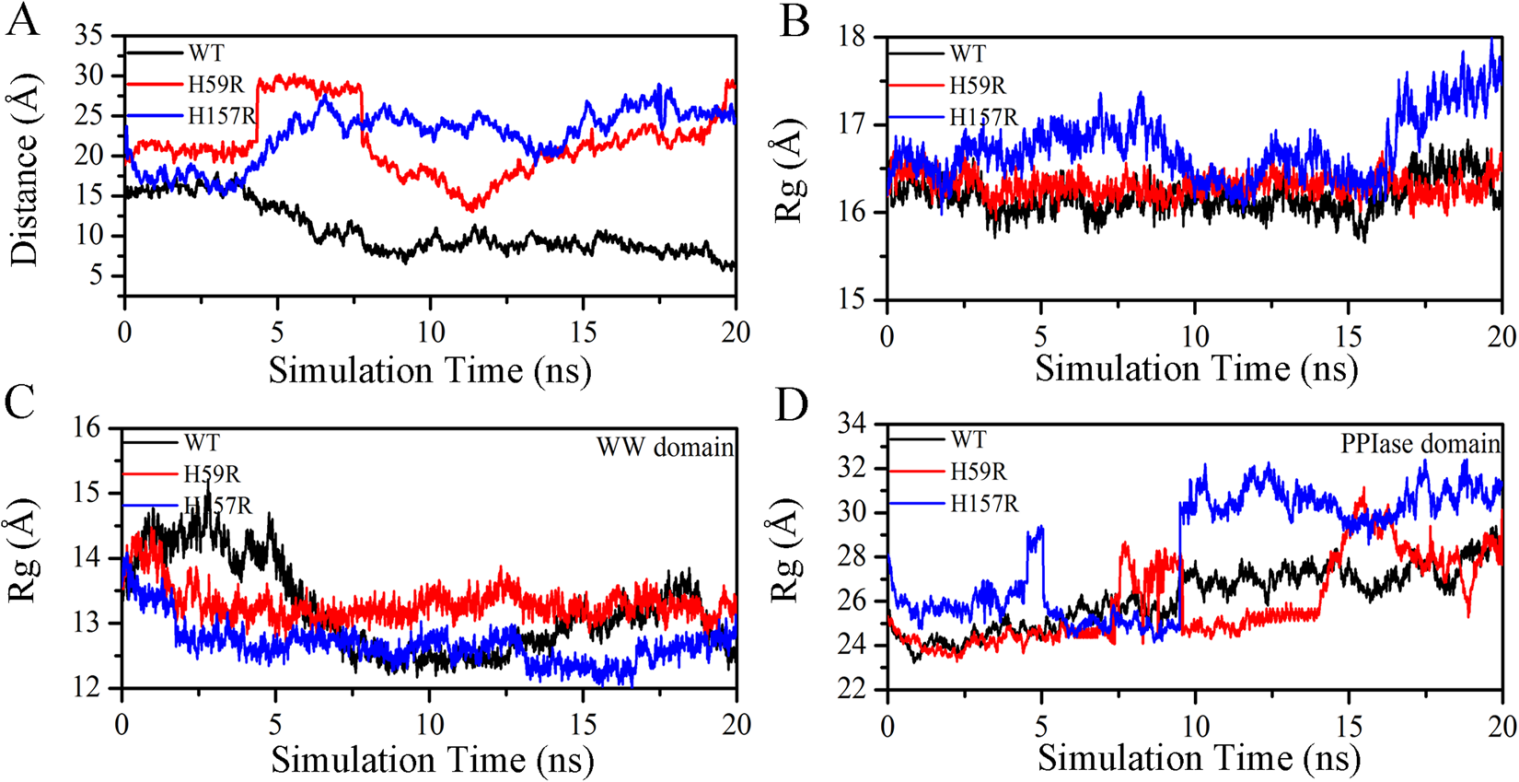
The distance and Rg result in Pin1-WT, H59R and H157R as a function of 20 ns MD simulation. (**A**) The distance between the WW and PPIase domain as a function of 20 ns MD simulation. (**B**–**D**) Represented Rg of the overall structure, PPIase and WW domain, respectively.

Generally, the function of the WW domain is mainly to identify the substrate, while the PPIase domain is the active center to catalyze substrate^11,30^. It is very meaningful to evaluate the distance between the WW and PPIase domain for analyzing the structure and function of Pin1. In Fig. 8A, the distance values of H59R and H157R were significantly higher than Pin1-WT after 5 ns MD simulations. Similarly, the average distance values of H59R (21.51 Å) and H157R (22.63 Å) were about the twofold increase, compared with Pin-WT (10.94 Å). Combined with the above results of RLS spectra, the RLS intensity of Pin1-WT at 450 nm was less than H59R and H157R, indicating that their sizes increase in the unfolding process. Therefore, we speculated that the histidine mutations may cause that the WW domain moves away from the PPIase domain in Pin1.

The radius of gyration (Rg), which is defined as a radial distance of atoms from the center of mass, usually estimate the compactness of protein^28^ (Fig. 8B–D). Obviously, the Rg value of global structure for H157R was greater than the others, suggesting that residue H157 may have an influence on the compactness of Pin1 (Fig. 8B and Table S4). The further analysis has shown that Rg values of the WW domain for Pin1-WT and mutants did not change significantly (Fig. 8C and Table S4). However, the Rg values of the PPIase domain were more sensitive to mutants, indicating that residues His59 and His157 mutations may result in a decrease in the compactness of the PPIase domain. (Fig. 8D and Table S4).

### Analysis of hydrogen bonds of the dual-histidine motif in the Pin1-WT, H59R and H157R

Previous studies have shown that a dual histidine motif is composed of residues His59 and His157 and they form a hydrogen bond network with residues Cys113 and Thr152^15,16^ (As seen in Fig. 1B). Therefore, the hydrogen bonds from the hydrogen bond network were calculated by molecular dynamic (MD) simulation method. Meanwhile, the backbone hydrogen bonds also were calculated by VMD1.9.2 program to estimate overall structural stability^31^. As seen in Table S4, compared with Pin1-WT, the average numbers of the backbone hydrogen bonds of H59R and H157R were decreased about two hydrogen bonds. Interestingly, the two missing hydrogen bonds located possibly at dual histidine motif owing to residues His59 and His157 mutations resulted in that the hydrogen bond network has been destroyed.

**Figure 9:**
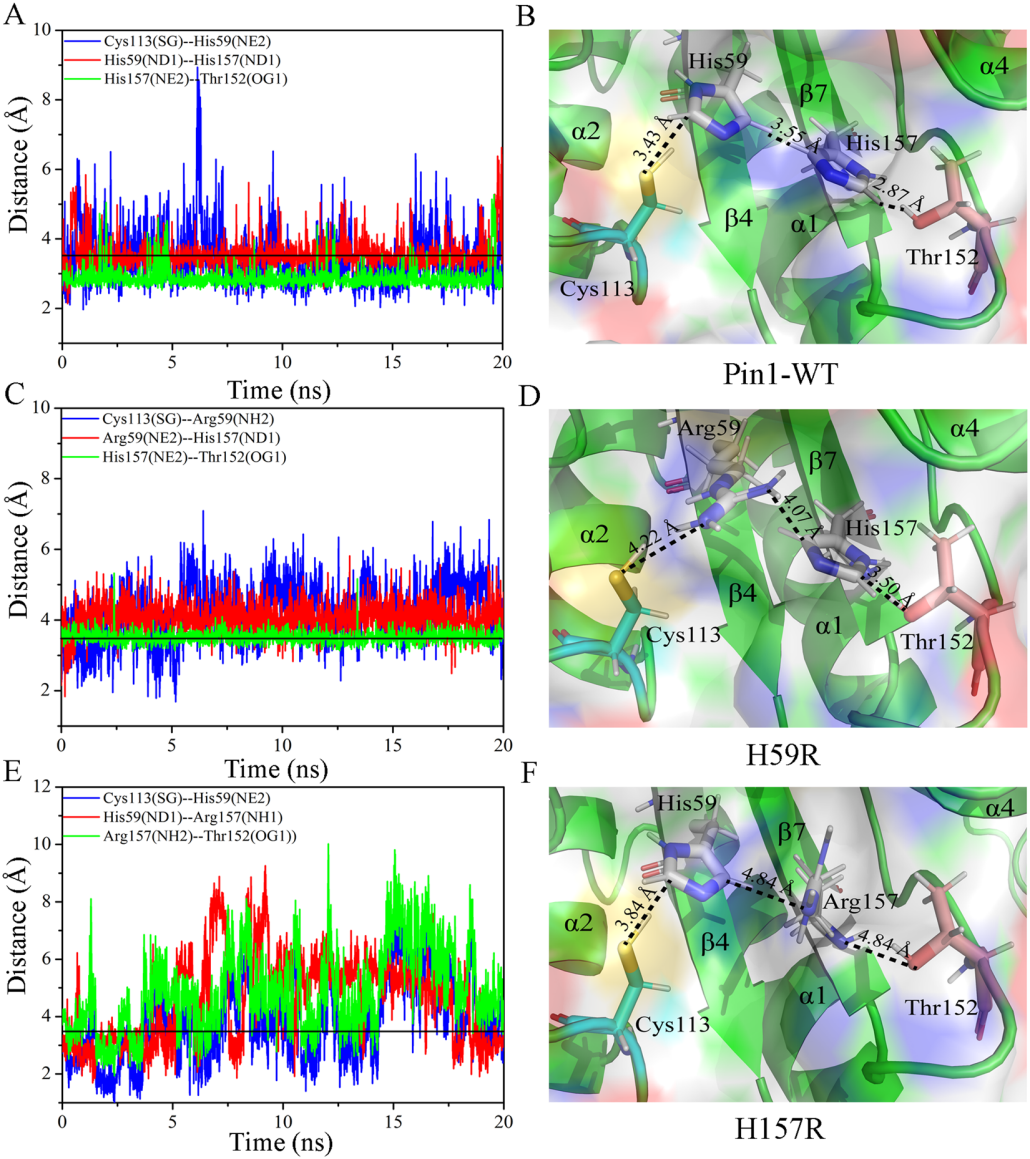
The distance between dual-histidine motif in Pin1-WT, H59R and H157R as a function of 20 ns MD simulation. (**A**,**B**) Represent the distance and model of the dual-histidine motif in Pin1-WT, respectively. (**C**,**D**) Represent the distance and model of the dual-histidine motif in H59R, respectively. (**E**,**F**) Represent the distance and model of the dual-histidine motif in H157R, respectively. The black line represents the cut-off distance of hydrogen bond as 3.5 Å.

We calculated the distances changes in the hydrogen bond network between partial atom pairs along with the 20 ns simulation time^32^. The Cys113: SG forms a hydrogen bond with His59: NE2, His59: ND1 forms a hydrogen bond with His157: ND1, and His157:NE2 forms a hydrogen bond with Thr152: OG1. In crystal structure of Pin1 (PDB code: 1PIN), the distance between Cys113: SG and His59: NE2 is 3.3 Å, it was consistent with the average distance of Pin1-WT (3.4 Å) and less than H59R (4.2 Å) and H157R (3.8 Å) along with MD simulation (Fig. 1B, Fig. 9 and Table S5). Moreover, the hydrogen bond occupancy between Cys113: SG and His59:NE2 was very short in these histidine mutants (Table S5). For the distance between His59:ND1 and His157:ND1, the crystal distance was observed about 2.9 Å, however, the average distance was 3.5 Å (WT-Pin1), 4.1 Å (H59R), 4.8 Å (H157R) along with MD simulation, respectively (Fig. 9 and Table S5). Interestingly, the occupancy of H59R between His59:ND1 and His157:ND1 was less than the others. Moreover, the distance between His157:NE2 and Thr152:OG1 was about 2.7 Å in the crystal structure, whereas the average distance and occupancy of H157R (4.8 Å and 19.1%) were significantly different from Pin-WT (2.9 Å and 96.6%) and H59R (3.5 Å and 63.1%), respectively (Table S5). These results illustrated that the hydrogen bond network was destroyed in the dual histidine motif by residues His59 and His157 mutations.

## Discussion and Conclusion

Pin1, a peptidyl-prolyl *cis-trans* isomerase, plays a key role in tumorigenesis and Alzheimer’s disease (AD)^3,33^. Several clinical trials have reported that Pin1 overexpression is closely related to multiple malignant cancers^7,8,34–36^, such as the colon, ovarian, cervical, endometrial, breast, prostatic and liver cancer. Whereas Pin1 inactivation is a potential cause of Alzheimer’s disease^9,33^. Up to now Pin1 exhibits a unique characteristic to specifically isomerize *cis/trans* conformation for p-Ser/Pro or p-Thr/Pro motifs, facilitating kinds of dephosphorylation pathway^3,37^. Isomerization of these motifs occurs in a modular fashion: the WW domain firstly recognizes them and then catalyzes them through the catalytic PPIase domain^38^.

A dual histidine motif, which consists of residues His59 and His157, forms a hydrogen bond network with residues Cys113 and Thr152 in the active site of the PPIase domain^39,40^. Zhou *et al*. and Behrsin *et al*. illustrated that mutants H157A/L/N still have partially activities^6,16^. Notably, Bailey *et al*. revealed that mutants H59X (X = A/N/S) and H157X (X = L/A/N/F/S) support partly viability, suggesting that the dual histidine motif has a structural role instead of catalytic function^15^. Moreover, Bailey *et al*. indicated that residue His157 is less important for enzyme function than His59^15^. Tossavainen *et al*. also reported the residue His59 plays a key role in the correct folding of the PPIase domain of PrsA from *Bacillus subtilis*^41^.

Exploring the structure and function of the dual histidine motif is necessary to understand the mechanism of the structural stability in the active site of Pin1 PPIase domain. Therefore, the H59R and H157R mutants were constructed for the following reasons: (1) Both arginine and histidine are basic amino acids with similar chemical properties; (2) Arginine mutants have not been studied and reported so far. Interestingly, my colleague measured the enzyme activity of Pin1-WT, H59R and H157R according to Behrsin *et al*.^16^ research, data not published, and the result suggested that two mutants have still partially activity (about 50%).

In the present work, the structural stability of wild-type and histidine mutants in Pin1 were extensive studies by thermal, acidic, chemical stability and MD simulation. Thermal denaturation revealed that both His59 and His157 are not sensitive to the lower temperatures, while His59 is more sensitive to the higher temperatures than His157, suggesting that His59 is possibly more important to maintain structural stability than His157. Wang *et al*. used site-directed mutagenesis to construct tryptophan mutants, including W11L, W34L and W73L, and illustrated that similar phenomenon about mutations reduce thermal stability^11^. Similarly, Bailey *et al*. also suggested that mutant H59L is less stable at higher temperatures, at least 50 °C, than H157L^16^. Acidic denaturation implied that influences of both residues His59 and His157 to acidic stability were difference from Pin1-WT. According to the crystal structure^14,29^, residue His59 is spatially closer to Cys113, which is considered as a critical catalytic residue in Pin1, than His157, indicating that it may play a role in catalysis. This may be one of the reasons that their acid stability is different. Moreover, the ANS and RLS spectra indicated that the histidine residues had little effect on the acidic pH-induced hydrophobic change and aggregation. One of the possible reasons is that a single amino acid mutation has little effect on the overall hydrophobicity and aggregation of the protein. Obviously, chemical denaturation suggested that both residues His59 and His157 contributed the most to the chemical stability of Pin1. Previous laboratory studies have indicated that key residue mutations cause a decrease in chemical stability and are more prone to unfolding^11^.

MD simulation can provide an atomic-level information for protein structures, which is difficult to obtain from traditional experiments. RMSD values suggested the histidine mutations mainly affect the structural stability of the PPIase domain rather than the WW domain. Similarly, RMSF values implied that residues His59 and His157 primarily affect the flexibility of the active center in the PPIase domain. It is well known that residues His59 and His157 are located in adjacent antiparallel β chains of the PPIase domain, so their mutations do not affect the WW domain^15,29^. Meanwhile, these results further indicated that residues His59 and His157 play an important role in stabilizing the PPIase domain. Obviously, our results have shown that the hydrogen bond network in the dual histidine motif was destroyed by residue His59 and His157 mutations. Chang *et al*. reported that mutant C113D destroyed the hydrogen bond network, leading to the instability of the catalytic tetrads (Cys113 -His59-His157-Thr152 motif)^42^. Barman *et al*. illustrated that the hydrogen bond network was rearranged by the protonation of residue Cys113, with the switching of the tautomeric states of the dual histidine motif^43^. Similarly, Wang *et al*. demonstrated that residues Cys113 and Ser138 modulate the hydrogen bond network dynamics due to the allosteric breakage of the hydrogen bond within the dual histidine motif in the PPIase domain^39,40^. These results suggested that the hydrogen bond network plays a very important role in stabilizing the PPIase domain, and a single residue mutation, including residues Cys113, His59, His157, and Thr152, has an effect on its stability. All in all, this work is based on the research of Bailey *et al*.^15^ to reveal deeply the mechanism by which residues His59 and His157 maintain the stability of the PPIase domain.

## Methods

### Preparation of Pin1-WT and mutants

The recombinant plasmids pET-19b-Pin1, pET-19b-H59R and pET-19b-H157R were expressed in *E. coli* BL21, which were induced with a specific condition (0.5 nM, 30 °C, 4 h)^11^. The interest proteins were purified by Ni-NAT Sepharose on an AKTA FPLC^30^. His-tag was excised by enterokinase and then separated through Amicon Ultra-15 centrifugal filter. The purity and concentration were detected by SDS-PAGE and Bradford assay, respectively^44^.

### Measurements of fluorescence spectra

The fluorescence spectra were measured on a F-4500 fluorescence spectrophotometer (Hitachi) with λ_ex_ = 295 nm (310–400 nm), which were set as 5 nm excitation and 10 nm emission slit^11,30^. The ANS (8-anilino-1-naphthalenesulfonic acid) fluorescence spectra were recorded with λ_ex_ = 380 nm (400–600 nm), setting as 5 nm excitation and 5 nm emission slit, respectively^22,45^. The RLS (Resonance light scattering) fluorescence spectra were recorded with Δλ = 0 nm (400–600 nm), setting as 2.5 nm excitation and 2.5 nm emission slit, respectively^24^. The concentration of Pin1-WT and mutants set to 5 μM.

To avoid the inner filter effect, the fluorescence intensities were corrected using the following relationship^46,47^:

1 $${{\rm{F}}}_{{\rm{cor}}}={{\rm{F}}}_{{\rm{obs}}}\times {{\rm{e}}}^{({{\rm{A}}}_{{\rm{ex}}}+{{\rm{A}}}_{{\rm{em}}})/2}$$

where *F*_*cor*_ and *F*_*obs*_ are the corrected fluorescence intensity and observed fluorescence intensity, respectively. *A*_*ex*_ and *A*_*em*_ represent the absorption of the excitation and the emission wavelength, respectively.

### Measurements of far-UV circular dichroism (CD) spectra

The far-UV CD spectra were recorded between 190 to 260 nm in 2 mm cuvettes by an Aviv Model 400 circular dichroism spectrophotometer (AVIV)^28,48^. The solvent spectrum was subtracted and the CDNN software was used to calculate the content of the secondary structure^11^. The concentration of Pin1-WT and mutants set to 10 μM.

### Thermal denaturation analysis of Pin1-WT and mutants

Fluorescence and far-UV CD measurements were implemented to assess the impacts of the histidine mutations on the thermal stability of Pin1^11,20,21^. Therefore, for fluorescence experiments, the thermal stability of Pin1-WT and mutants were performed from 20 to 95 °C at an interval of 5 °C using peltier temperature controller. The samples (5 μM) of Pin1-WT and mutants were incubated a given conditions (20 mM Tris-HCl, pH 7.0) for 5 min. For far-UV CD experiments, the samples (10 μM) were incubated for 5 min from 20 to 80 °C, and the ellipticity at 208 nm was recorded.

In the unfolding studies, the fraction of Pin1-WT and mutants at each temperature was calculated using the following equation^11,19^:

2 $${{\rm{f}}}_{u}=({F}_{obs}-{F}_{n})/({F}_{d}-{F}_{n})$$

where *f*_*u*_ is the fraction of unfolded Pin1-WT and mutants at a given temperature, *F*_*obs*_ is the fluorescence intensity or CD signal intensity at the temperature, *F*_*n*_ and *F*_*d*_ are the fluorescence intensity or CD signal intensity of the native and denatured protein, respectively. *T*_*m*_, a temperature of the denaturation process at the midpoint, was calculated by the plotting *f*_*u*_ against temperature^11,23,49^.

Based on a two-state model, the apparent Gibbs free energy (*ΔG*_*u*_) was calculated using the following equation at each temperature^19,50^:

3 $${{\rm{\Delta }}G}_{{\rm{u}}}=-\,{{\rm{RTlnK}}}_{{\rm{eq}}}$$

where *R* and *T* are the ideal gas constant and specific temperature, respectively. *K*_*eq*_ is the fraction of unfolded protein at each temperature. The apparent Gibbs free energy (*ΔG*_*u*_) is related to the free energy of unfolding $(\Delta {{G}}^{{{H}}_{{2}}{O}})$ by the following equation^23,51^:

4 $${{\rm{\Delta }}G}_{{\rm{u}}}={{\rm{\Delta }}G}^{{{\rm{H}}}_{{\rm{2}}}{\rm{O}}}-{\rm{mT}}$$

where *m* is the experimental measure of the dependence of *ΔG*_*u*_ on temperature.

### Acidic denaturation analysis of Pin1-WT and mutants

The acidic-pH induced denaturation of Pin1-WT and mutants were evaluated by fluorescence and far-UV CD measurements^24^. The solution of Pin1-WT and mutants were added to the different acidic pH buffer. The ANS fluorescence spectra were carried out to assess the extent of hydrophobic surface exposure in Pin1-WT and mutants, and the fluorescence intensity at 488 nm was recorded^24^. The RLS fluorescence spectra were implemented to assess the aggregation of Pin1-WT and mutants, and the signal intensity at 450 nm was recorded to gauss curves in origin 8.5 software.

### Chemical denaturation analysis of Pin1-WT and mutants

The chemical denaturation of Pin1-WT and mutants were assessed by fluorescence and far-UV CD experiments. The samples were incubated at GdnHCl solution of different concentrations for 2 h at room temperature. The unfolding constants for Pin1-WT and mutants were calculated according to the following equation^19,50^:

5 $${{\rm{K}}}_{{\rm{ui}}}={{\rm{P}}}_{{\rm{t}}}{{\rm{f}}}_{{\rm{u}}}{/[{\rm{P}}}_{{\rm{t}}}(1-{{\rm{f}}}_{{\rm{u}}}{)]}^{{\rm{n}}}$$

where *K*_*ui*_ is unfolding constant, *P*_*t*_ is the molecular concentration of protein when all the protein is folded, *f*_*u*_ is the fraction of unfolded, *n* is the number of the chain formed after denaturation.

The free energy (*ΔG*_*ui*_) of folding at any given concentration was evaluated from the following equation^19,50^:

6 $${{\rm{\Delta }}G}_{{\rm{ui}}}=-\,{\rm{RT}}\,\mathrm{ln}\,{{\rm{K}}}_{{\rm{ui}}}$$

where *R* and *T* are the ideal gas constant and specific temperature, respectively. Free energy of protein unfolding (*ΔG*_0_) was obtained from a plot of *ΔG*_*ui*_ (y) as a function of GdnHCl concentrations (x) where y intercept equals to the *ΔG*_0_ value. Similarly, the values of unfolding kinetics rate constants (*K*_*obs*_) was determined from a half-Chevron plot of *K*_*ui*_ (y) as a function of GdnHCl concentrations (x) where y intercept equals to the *K*_*obs*_ value.

### Molecular dynamics simulation

The crystal structure of Pin1 was downloaded from Protein Data Base (PDB code: 1PIN)^14,52^. The water molecules, ions and ligands were removed and missing residues were added by the Modeller 9.16 software^53^. Two mutants H59R and H157R were constructed by using Pymol 1.X program^54^.

The MD simulations of Pin1-WT and mutants were performed by Gromacs 4.6.5 with Gromacs 96–53a6 force filed^55–57^. Before the formal simulation, the energy minimization was carried out for all systems^22,27^. Firstly, steepest descent method was adopted to energy minimization of 1000 steps and then heated to 300 K in vacuum^27^. Secondly, all the systems were immersed in a cubic box and solvated with SPC water patterns using a solute-box distance of 1.2 nm^27^. To neutralize the system, Na^+^ and Cl^−^ ions were inserted in the solute-box^58,59^. Thirdly, all systems were further performed energy minimization of 100 ps in the NVT ensemble, and subsequently equilibrated for 100 ps in the NPT ensemble^30^. All systems were performed to 20 ns simulation in the NPT ensemble. The temperature and pressure were controlled at 300 K and 1 atm by the methods of Velocity-Rescaling and Parrinello-Rahman, respectively^60^. The short-range interactions for cutoff and switch distance were set to 12 Å and 10 Å, respectively^61^. The long-range electrostatic interaction was described by Particle mesh Ewald (PME) method^62^. LINCS (Linear Constraint Algorithm) method was used to constrain the hydrogen bonds^63^.

## Supplementary information

Supplementary material
