# Supplementary material for: Insight into the structural stability of wild-type and histidine mutants in Pin1 by experimental and computational methods
Source: Sci Rep. 2019 Jun 10;9:8413. doi: 10.1038/s41598-019-44926-5 (PMC6557836; doi:10.1038/s41598-019-44926-5)
Supplement: Supplementary file 1 — Supplementary material [file 41598_2019_44926_MOESM1_ESM.docx]

**Supplement files**

#

# **Insight into** **the structural stability of wild-type and histidine mutants in Pin1 by** **experimental and computational methods**

Wang Wang^+^, Lei Xi^+^, Xiuhong Xiong, Xue Li, Qingyan Zhang, Wentao Yang, Linfang Du

*Key Laboratory of Bio-resources and Eco-environment of the Ministry of Education, College of Life Sciences, Sichuan University, Chengdu 610064, PR of China*

*Corresponding author: Tel.: +86 028 85415008; fax: +86 028 85415300.

E-mail: dulinfang@scu.edu.cn (L.F. Du)

^+^ These authors contributed equally to this work.

**Table captions**

**Table S1.** The comparison of secondary structure of Pin1-WT, H59R，H157R at various temperature.

**Table S2.** The comparison of secondary structure of Pin1-WT, H59R, H157R at various acidic pH.

**Table S3.** The comparison of secondary structure of Pin1-WT, H59R, H157R at various concentration of GdnHCl.

**Table S4.** Comparison of the various properties of Pin1-WT, H59R and H157R.

**Table S5.** The occupancy of hydrogen bonds forming at the dual-histidine motif.

| Sample | T (℃) | 20 | 30 | 40 | 50 | 60 | 70 | 80 |
| --- | --- | --- | --- | --- | --- | --- | --- | --- |
| Pin1-WT | α-Helix (%) | 19.66 | 19.40 | 19.10 | 17.55 | 17.00 | 16.82 | 16.49 |
|  | β-Sheet (%) | 23.82 | 24.03 | 24.25 | 25.56 | 26.17 | 26.26 | 26.52 |
|  | β-Turn (%) | 17.72 | 17.59 | 17.65 | 18.09 | 18.35 | 18.44 | 18.37 |
|  | coils (%) | 38.81 | 38.98 | 39.00 | 38.79 | 38.49 | 38.49 | 38.62 |
| H59R | α-Helix (%) | 20.01 | 19.98 | 19.64 | 17.68 | 17.64 | 16.93 | 16.34 |
|  | β-Sheet (%) | 24.02 | 24.26 | 24.51 | 25.15 | 25.13 | 25.79 | 26.07 |
|  | β-Turn (%) | 17.75 | 17.74 | 17.84 | 18.42 | 18.57 | 18.76 | 18.87 |
|  | coils (%) | 38.22 | 38.32 | 38.01 | 38.84 | 38.66 | 38.51 | 38.71 |
| H157R | α-Helix (%) | 19.76 | 19.48 | 19.26 | 17.62 | 17.03 | 16.63 | 16.22 |
|  | β-Sheet (%) | 23.97 | 24.20 | 24.47 | 25.65 | 26.15 | 26.81 | 27.05 |
|  | β-Turn (%) | 17.75 | 17.77 | 17.75 | 18.25 | 18.25 | 18.31 | 18.36 |
|  | coils (%) | 38.52 | 38.55 | 38.52 | 38.48 | 38.57 | 38.25 | 38.37 |

**Table S1.** The comparison of secondary structure of Pin1-WT, H59R, H157R at various temperature.

| Sample | pH | 7 | 6 | 5 | 4 | 3 | 2 | 1 |
| --- | --- | --- | --- | --- | --- | --- | --- | --- |
| Pin1 | α-Helix (%) | 20.12 | 20.23 | 19.85 | 19.87 | 18.07 | 15.37 | 13.17 |
|  | β-Sheet (%) | 24.07 | 23.53 | 24.03 | 24.63 | 25.88 | 24.88 | 24.38 |
|  | β-Turn (%) | 17.55 | 17.91 | 18.01 | 17.16 | 17.84 | 17.04 | 16.94 |
|  | coils (%) | 38.26 | 38.34 | 38.11 | 38.34 | 38.22 | 42.72 | 45.51 |
| H59R | α-Helix (%) | 19.14 | 18.81 | 18.09 | 17.31 | 17.03 | 15.25 | 13.22 |
|  | β-Sheet (%) | 25.00 | 25.72 | 26.04 | 26.57 | 26.87 | 24.8 | 24.59 |
|  | β-Turn (%) | 18.59 | 18.16 | 17.90 | 19.02 | 19.08 | 17.04 | 16.02 |
|  | coils (%) | 37.36 | 37.31 | 37.96 | 37.10 | 37.02 | 42.91 | 46.17 |
| H157R | α-Helix (%) | 19.76 | 19.43 | 18.99 | 18.00 | 17.33 | 16.01 | 13.10 |
|  | β-Sheet (%) | 23.97 | 24.31 | 24.63 | 24.98 | 25.41 | 24.88 | 24.38 |
|  | β-Turn (%) | 17.75 | 17.91 | 17.90 | 18.10 | 18.45 | 17.14 | 16.33 |
|  | coils (%) | 38.52 | 38.35 | 38.48 | 38.92 | 38.81 | 41.97 | 46.28 |

**Table S2.** The comparison of secondary structure of Pin1-WT, H59R, H157R at various acidic pH.

| Sample | GdnHCl | 0 | 1 | 2 | 3 | 4 | 5 | 6 | 7 |
| --- | --- | --- | --- | --- | --- | --- | --- | --- | --- |
| Pin1 | α-Helix (%) | 20.59 | 17.20 | 14.53 | 13.42 | 12.90 | 12.46 | 10.88 | 10.46 |
|  | β-Sheet (%) | 22.63 | 24.46 | 26.12 | 26.93 | 27.32 | 27.51 | 28.73 | 29.12 |
|  | β-Turn (%) | 17.90 | 18.01 | 18.08 | 18.10 | 18.04 | 18.06 | 18.02 | 18.02 |
|  | coils (%) | 38.87 | 40.32 | 41.18 | 41.63 | 41.74 | 41.89 | 42.37 | 42.48 |
| H59R | α-Helix (%) | 19.34 | 16.31 | 14.09 | 13.21 | 12.15 | 12.24 | 10.16 | 10.97 |
|  | β-Sheet (%) | 23.28 | 25.00 | 26.46 | 27.10 | 27.79 | 27.73 | 29.36 | 28.76 |
|  | β-Turn (%) | 17.97 | 18.09 | 18.13 | 18.12 | 18.05 | 18.07 | 18.00 | 18.03 |
|  | coils (%) | 39.41 | 40.6 | 41.32 | 41.66 | 42.01 | 41.97 | 42.56 | 42.32 |
| H157R | α-Helix (%) | 19.23 | 15.96 | 13.86 | 12.89 | 12.46 | 11.51 | 10.78 | 9.05 |
|  | β-Sheet (%) | 23.35 | 25.22 | 26.60 | 27.30 | 27.51 | 28.21 | 28.85 | 30.27 |
|  | β-Turn (%) | 17.95 | 18.08 | 18.05 | 18.11 | 18.06 | 18.01 | 17.99 | 17.86 |
|  | coils (%) | 39.47 | 40.74 | 41.49 | 41.79 | 41.89 | 42.19 | 42.38 | 42.82 |

**Table S3.** The comparison of secondary structure of Pin1-WT, H59R, H157R at various concentration of GdnHCl.

| System | WT | H59R | H157R | ΔH59R | ΔH157R |
| --- | --- | --- | --- | --- | --- |
| RMSD (Å) | 2.86 ± 0.39 | 2.97 ± 0.39 | 3.03 ± 0.32 | 0.11 | 0.17 |
| RMSD of WW (Å) | 2.55 ± 0.24 | 2.59 ± 0.27 | 2.65 ± 0.26 | 0.04 | 0.10 |
| RMSD of PPIase (Å) | 3.54 ± 0.41 | 3.67 ± 0.38 | 3.85 ± 0.38 | 0.13 | 0.31 |
| RMSF (Å) | 1.04 ± 0.54 | 1.63 ± 0.81 | 1.96 ± 0.83 | 0.59 | 0.92 |
| RMSF of WW (Å) | 1.29 ± 0.73 | 2.28 ± 1.27 | 2.63 ± 0.84 | 0.99 | 1.34 |
| RMSF of PPIase (Å) | 0.87 ± 0.31 | 1.41 ± 0.44 | 1.62 ± 0.56 | 0.54 | 0.75 |
| Rg (Å) | 13.21 ± 0.70 | 13.31 ± 0.27 | 12.68 ± 0.32 | 0.10 | -0.53 |
| Rg of WW (Å) | 16.18 ± 0.19 | 16.31 ± 0.13 | 16.74 ± 0.36 | 0.13 | 0.56 |
| Rg of PPIase (Å) | 26.19 ± 1.41 | 26.34± 2.06 | 28.26 ± 2.61 | 0.15 | 2.07 |
| Distance (Å) | 10.94 ± 3.61 | 21.51± 4.63 | 22.63 ± 3.26 | 10.57 | 11.69 |
| Hydrogen bonds | 36.09 ± 5.19 | 33.99 ± 5.04 | 33.49 ± 4.88 | -2.10 | -2.60 |

**Table S4.** Comparison of the various properties of Pin1-WT, H59R and H157R. ΔH59R and ΔH157R represented the values of (H59R-WT) and (H157R-WT), respectively.

| Sample | Donor | Acceptor | Occupancy (%) | Average distance (Å) |
| --- | --- | --- | --- | --- |
| Pin1-WT | Cys113:SG | His59:ND1 | 64.68 ± 1.42 | 3.43 ± 0.78 |
|  | His59:NE2 | His157:NE2 | 64.96 ± 5.20 | 3.55 ± 0.40 |
|  | His157:ND1 | Thr152:OG1 | 96.60 ± 2.11 | 2.87 ± 0.32 |
| H59R | Cys113:SG | Arg59:NH2 | 20.83 ± 0.92 | 4.22 ± 0.83 |
|  | Arg59:NH1 | His157:NE2 | 9.02 ± 1.02 | 4.07 ± 0.43 |
|  | His157:ND1 | Thr152:OG1 | 63.09 ± 0.67 | 3.50 ± 0.16 |
| H157R | Cys113:SG | His59:ND1 | 48.38 ± 3.12 | 3.84 ± 1.44 |
|  | His59:NE2 | Arg157:NH1 | 36.33 ± 0.68 | 4.84 ± 1.50 |
|  | Arg157:NH2 | Thr152:OG1 | 19.09 ± 0.10 | 4.84 ± 1.44 |

**Table S5.** The occupancy of hydrogen bonds forming at the dual-histidine motif.
